# Virucidal efficacy of laundry sanitizers against SARS-CoV-2 and other coronaviruses and influenza viruses

**DOI:** 10.1038/s41598-022-08259-0

**Published:** 2022-03-28

**Authors:** M. Khalid Ijaz, Raymond W. Nims, Julie McKinney, Charles P. Gerba

**Affiliations:** 1 Global Research and Development for Lysol and Dettol, Reckitt Benckiser LLC, One Philips Parkway, Montvale, NJ 07645 USA; 2 RMC Pharmaceutical Solutions, Inc., 1851 Lefthand Circle, Suite A, Longmont, CO 80501 USA; 3 Department of Environmental Sciences, University of Arizona, Tucson, AZ USA

**Keywords:** Antimicrobials, Applied microbiology

## Abstract

The clothes laundering process affords numerous opportunities for dissemination of infectious virus from contaminated clothing to appliance surfaces and other household surfaces and eventually to launderer’s hands. We have explored the efficacy of laundry sanitizers for inactivating coronaviruses and influenza viruses. Virucidal efficacy was tested using standardized suspension inactivation methods (EN 14476) or hard-surface inactivation methods (ASTM E1053-20) against SARS-CoV-2, human coronavirus 229E (HCoV 229E), influenza A virus (2009-H1N1 A/Mexico), or influenza B virus (B/Hong Kong). Efficacy was measured in terms of log_10_ reduction in infectious virus titer, after 15 min contact time (suspension studies) or 5 min contact time (hard surface studies) at 20 ± 1 °C. In liquid suspension studies, laundry sanitizers containing *p*-chloro-*m*-xylenol (PCMX) or quaternary ammonium compounds (QAC) caused complete inactivation (≥ 4 log_10_) of HCoV 229E and SARS-CoV-2 within 15 min contact time at 20 ± 1 °C. In hard surface studies, complete inactivation (≥ 4 log_10_) of each coronavirus or influenza virus, including SARS-CoV-2, was observed following a 5-min contact time at 20 ± 1 °C. Respiratory viruses may remain infectious on clothing/fabrics and environmental surfaces for hours to days. The use of a laundry sanitizer containing microbicidal actives may afford mitigation of the risk of contamination of surfaces during handling of the laundry and washing appliances (i.e., washer/dryer or basin), adjacent surfaces, the waste water stream, and the hands of individuals handling clothes contaminated with SARS-CoV-2, influenza viruses, or other emerging enveloped viruses.

## Introduction

Laundry sanitizers have been introduced to commerce to enhance the bactericidal and virucidal efficacy of the clothes-washing process. It could be argued that laundry detergent, in association with elevated water temperatures, has sufficient microbicidal efficacy that an additional agent (i.e., the sanitizer) is not required. There are several factors to consider, however, when addressing this issue. The clothes-washing process is complex, and consists of multiple steps capable of reducing pathogen load^1,2^. These steps include: (1) removal, through the action of the detergent and the water rinse; (2) inactivation by the detergent; and (3) possible thermal inactivation by the water used for soaking and rinsing. From a virucidal point of view, it may be assumed that detergent inactivation should apply primarily to lipid-enveloped viruses^3,4^, while removal should apply to all viruses (i.e., both lipid-enveloped and well as non-enveloped). Extent of thermal inactivation will be dependent upon the temperature of the water used for the wash and rinse portions of the washing cycle, and upon the target virus. Usually, 40 °C or higher is recommended for eliminating bacterial and viral pathogens^2^. In the case of cold (20 to 23 °C)^5^ and warm water (≤ 40 °C)^2^ cycles, minimal inactivation attributable solely to heating (i.e., thermal inactivation alone, in the absence of detergent) of SARS-CoV-2 would be expected over the time course of a washing cycle^3,6,7^. Removal of non-inactivated virus simply transfers infectious virus from one location to another, possibly contaminating other surfaces and the waste-water stream^5^. The wastewater (gray water) stream may be reused in some households for landscape irrigation, flushing toilets or other purposes^8^. Another consideration is that some types of clothing can only be hand-washed and, in some regions of the world, hand-washing of clothing is the only option available^9^. Even in North America ~ 6% of laundry is still hand washed^9^. To reduce the risks from pathogens and for a higher level of assurance of interrupting the spread of highly pathogenic viruses via contaminating clothing and environmental surfaces associated with the clothes laundering process, the use of EPA-registered laundry sanitizers, surface hygiene agents, and hand hygiene agents may be warranted^10,11^. This is especially true during a viral outbreak such as the severe acute respiratory syndrome virus-2 (SARS-CoV-2) pandemic now being experienced and the emergence of mutational variants with increased morbidity or transmissibility (e.g., the Delta and Omicron variants).

**Figure 1:**
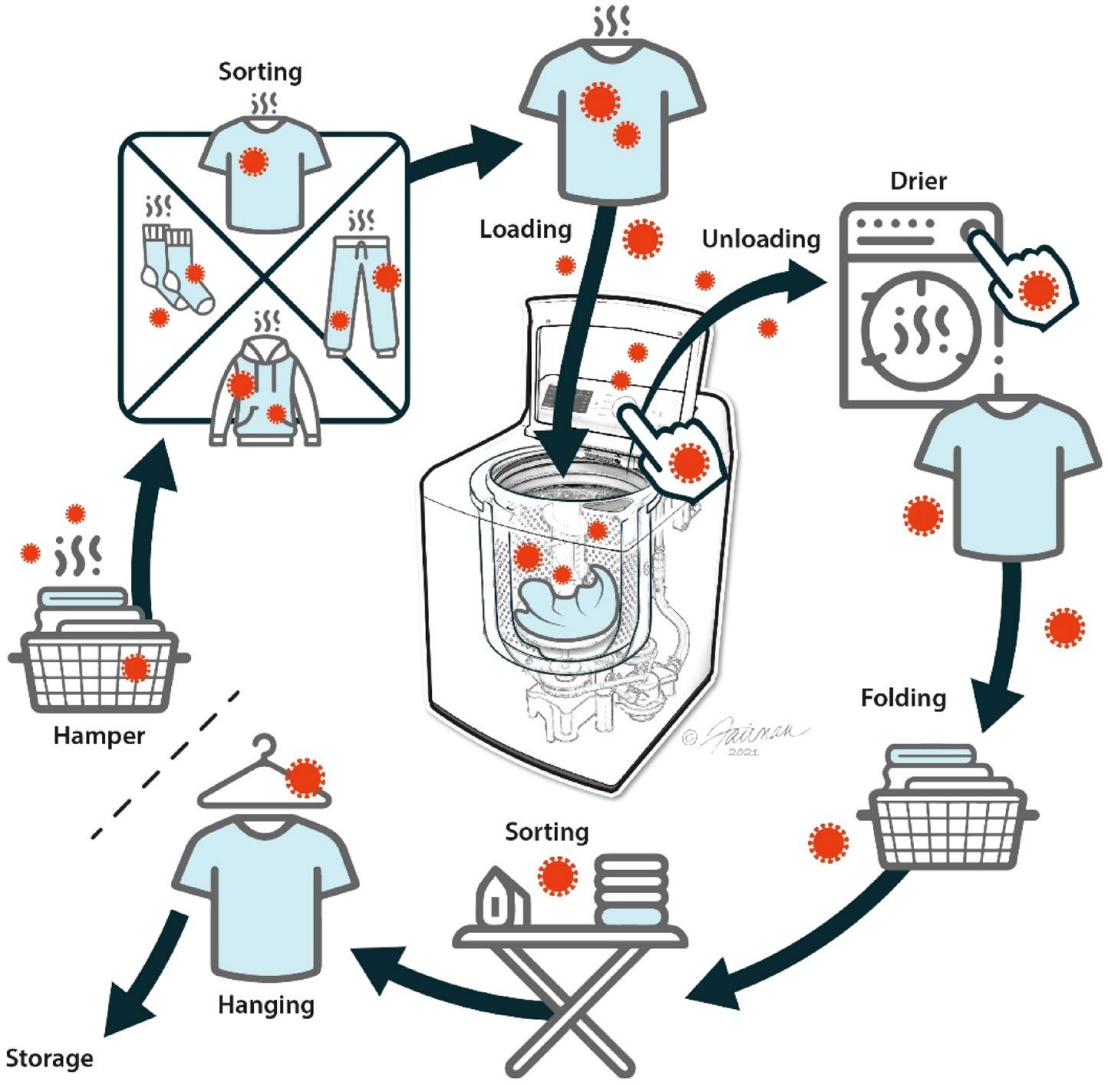
Schematic view of the machine clothes laundering process, indicating possible risk points for enveloped virus accumulation and cross-contamination. These cross-contaminations can potentially be mitigated by application of additional targeted hand/surface hygiene agents^26,33^.

A few marketed laundry sanitizing agents have been characterized as antibacterial. We were unable to identify reports of the ability of such products to inactivate viruses in general, or SARS-CoV-2, in particular. In the present study, we have examined the virucidal efficacy of a selection of formulated microbicidal active-containing laundry sanitizers against four enveloped viruses: coronaviruses, including the alphacoronavirus human coronavirus 229E (HCoV 229E) and the betacoronavirus SARS-CoV-2), and the orthomyxoviruses influenza A and B. The suspension testing methodology described in international standard EN 14,476:2013 + A2:2019^12^ and the hard surface testing methodology described in ASTM International E-1053-20^ 13^ were employed. As mentioned above, there are multiple opportunities for dissemination of virus during the laundering process, and not all of these are addressed by the actual efficacy for viral removal and inactivation by the detergent and water-based washing and rinsing process. Other risks may best be mitigated through use of additional hygiene agents, including possibly laundry sanitizers, surface hygiene agents, and hand hygiene agents^10,11^. The rationale for conducting both suspension and hard surface testing was that laundry sanitizers are intended not only to sanitize the washed clothes but also the surfaces of the washing machines exposed to potentially contaminated clothes/wash/rinse solutions (Fig. 1).

## Methods

### Challenge viruses, host cell lines, and reagents

Virucidal efficacy testing against alpha- and beta-coronaviruses and influenza viruses A and B was performed for commercially available laundry sanitizer products per standardized hard surface and suspension methods. Details on the challenge viruses and the host cell lines used for propagation of viral stocks and for in vitro cell-based infectivity assays are shown in Table 1. This table also indicates the culture media used for propagating the cells and the contract testing organizations that performed the virucidal efficacy testing.

**Table 1:** Viruses and detector cell lines used.

Species	Genus	Strain	Source	Cell line	Source	Description	Culture medium
2009-H1N1 Influenza A virus^a^	A	A/Mexico/4108/2009	CDC #2,009,712,192	MDCK	ATCC CCL-34	Canine kidney	DMEM + 2 μg/mL TPCK-trypsin
Influenza B virus^a^	B	B/Hong Kong/5/72	ATCC VR-823	MDCK	ATCC CCL-34	Canine kidney	DMEM + 2 μg/mL TPCK-trypsin
Human coronavirus^a^	Alphacoronavirus	HCoV 229E	ATCC VR-740	WI-38	ATCC CCL-75	Human lung	MEM + 2% FBS
Human coronavirus^b\^	Alphacoronavirus	HCoV 229E	ATCC VR-740	MRC-5	ATCC CCL-171	Human lung	MEM + 2% FBS
SARS-CoV-2^a,b^	Betacoronavirus	Isolate USA-WA1/2020	CDC, through BRI Resources	Vero E6	ATCC CRL-1586	African green monkey kidney	MEM + 2% FBS

*ATCC* American Type Culture Collection, *CDC* U.S. Centers for Disease Control and Prevention, *FBS* fetal bovine serum, *HCoV 229E* human coronavirus 229E, *MEM* minimal essential medium, *SARS-CoV-2* severe acute respiratory syndrome coronavirus 2, *TPCK* N-tosyl-L-phenylalanine chloromethyl ketone.

^a^Testing performed at Accuratus Lab Services, Eagan, MN, USA.

^b^Testing performed at Microbac Laboratories, Inc., Sterling, VA, USA.

### Standardized suspension efficacy testing methodology

Virucidal efficacy evaluations of laundry sanitizers against coronaviruses suspended in liquid matrices were conducted per EN 14476:2013 + A2:2019^12^. The challenge matrix in each case was cell culture medium containing an organic load. The microbicidal active ingredient concentrations in the products as tested, contact times, exposure temperatures, and the organic loads evaluated, are each indicated in Table 2. A brief description of the methodology follows: One-mL soil load at 10 × concentration was mixed with an equal volume of virus. Eight mL of formulated microbicidal active-containing laundry sanitizer, at concentration sufficient to achieve the final concentration listed in Table 2, were added. The resulting solutions were subjected to vortex mixing. The test solutions were held for the indicated contact times at 20 ± 1 °C. Following the exposure periods, the test solutions were immediately neutralized by adding ice-cold neutralizing agent, defined in Table 2, to stop the virucidal reactions. In certain cases, as indicated in Table 2, the neutralized samples were passed through a Sephadex LH-20 gel filtration column to reduce cytotoxicity to the detector cells used in assessing any residual infectious virus. Neutralized test solutions were serially ten-fold diluted in a dilution medium (culture medium; defined in Table 1) and inoculated onto host cells to assay for infectious virus titer using a 50% tissue culture infectious dose (TCID_50_) assay.

**Table 2:** Virucidal efficacy of laundry sanitizers tested per EN 14,476:2013 + A2:2019 on SARS-CoV-2 and HCoV 229E in suspension studies in the presence of an organic load.

Active ingredient and tested concentration	Temperature	Contact time (minutes)	Log_10_ reduction in infectious titer achieved^a^	
			Alpha-coronavirus	Beta-coronavirus
			HCoV 229E	SARS-CoV-2
PCMX (0.033% in 100 ppm AOAC hard water), 1:50 dilution of the product in hard water	20 ± 1 °C	15	≥ 5.2^b^	≥ 5.2^b^
QAC^d^ (0.055% in hard water), 1:42 dilution of the product	20 ± 1 °C	15	Not tested	≥ 5.0^c^
QAC^d^ (0.057% in hard water), 1:42 dilution of the product	20 ± 1 °C	15	Not tested	≥ 4.2^b^

*HCoV 229E* human coronavirus 229E, *PCMX* *p*-chloro-*m*-xylenol, *QAC* quaternary ammonium compound, *SARS-CoV-2* severe acute respiratory syndrome coronavirus 2, *w/v* weight to volume, *w/w* weight to weight.

^a^In all cases, a single replicate measurement was used to generate the data point.

^b^Neutralizer used: minimal essential medium + 10% fetal bovine serum; organic load: 0.3% bovine serum albumin + 0.3% erythrocyte solution.

^c^Cytotoxicity was reduced by passage of the virus/test substance through a Sephadex LH-20 gel filtration column; organic load: 0.3% bovine serum albumin.

^d^QAC included benzalkonium chloride and dialkyldimethylammonium chloride.

### Standardized hard surface efficacy testing methodology

Virucidal efficacy evaluations of laundry sanitizers against viruses experimentally deposited on a prototypic non-porous surface (glass) were conducted per ASTM E1053-20^13^. The microbicidal active ingredient concentrations, contact times, exposure temperatures, and the organic loads evaluated are indicated in Table 3. A brief description of the methodology follows: An aliquot of 0.4 mL of virus plus soil load was added onto a pre-sterilized 10-cm^2^ glass Petri dish and spread over the surface of the carrier. The virus was allowed to dry at ambient temperature. The laundry sanitizer under evaluation (2.0 mL) was added onto the dried viral film to completely cover the virus film. The carriers were then held for the indicated contact times at 20 ± 1 °C. Neutralizing agent (2.0 mL) was then added, and the viral inoculum/sanitizer/neutralizer mixture was scraped off the dish using a cell scraper. The neutralized test solutions were passed through a gel filtration column to reduce cytotoxicity to the host cells. The neutralized samples were serially ten-fold diluted in a dilution medium defined in Table 3 and inoculated onto detector cells to assay for infectious virus using the TCID_50_ assay.

**Table 3:** Virucidal efficacy of a laundry sanitizer tested per ASTM E1053-20 against coronaviruses and influenza viruses dried on a glass surface in the presence of a 5% fetal bovine serum organic load.

Active ingredient and tested concentration	Temperature (% RH)	Contact time (minutes)	Log_10_ reduction in infectious titer achieved^a^			
			2009-H1N1 Influenza A virus	Influenza B virus	Alpha-coronavirus	Beta-coronavirus
					HCoV 229E	SARS-CoV-2
QAC^d^ (0.08%) 1:28 of product in 400 ppm AOAC hard water	20 ± 1 °C (25–40%)	5	≥ 5.0, ≥ 5.0^b^	≥ 3.0, ≥ 3.0^b^	≥ 4.5, ≥ 4.5^b^	≥ 3.0, ≥ 3.0, ≥ 3.0^c^

*AOAC* Association of Official Analytical Chemists, *HCoV 229E* human coronavirus 229E, *QAC* quaternary ammonium compound, *SARS-CoV-2* severe acute respiratory syndrome coronavirus 2, *RH* relative humidity.

^a^Where multiple values are displayed, this reflects the testing of multiple independent lots of the test sanitizer. In all cases, a single replicate measurement was used to generate the data point.

^b^Cytotoxicity was reduced by passage of the virus/test substance through a Sephadex LH-20 gel filtration column.

^c^Neutralizer used: minimal essential medium + 10% fetal bovine serum + 0.5% polysorbate 80 + 0.5% lecithin.

^d^ BTC 8358 + Bardac 2080.

### Calculation of log_10_ reduction in titer, survival half-lives, and time required to reach fabric virus burdens below the estimated human infectious dose_50_ (ID_50_)

Virucidal efficacy data obtained from suspension inactivation and non-porous surface (glass) inactivation studies have been presented in terms of log_10_ reduction in titer of the virus, with titers being calculated using a 96-well plate cell infectivity assay. Scoring for viral titer was based on viral cytopathic effect (CPE) in the host cell monolayers. The results have been expressed in units of log_10_ tissue culture infectious dose_50_ per mL (TCID_50_/mL), calculated per Reed and Meunch^14^. Log_10_ reduction in titer values have been obtained by subtracting the post-treatment titers from the corresponding positive control titers. Limits of detection for the detection assays applied in some cases, due to residual cytotoxic effects of the formulated microbicidal active-containing laundry sanitizers following neutralization. Such limits of detection have been accounted for in determination of log_10_ reduction values.

Survival half-life (t½) values of viruses on experimentally contaminated fabric articles were reported or have been calculated from the reported data for SARS-CoV-2^6,15–19^ or influenza virus H1N1^20,21^. Biphasic linear regression plots (log_10_ titer *vs.* time) of the survival data were used to calculate the terminal survival half-lives (t½), as t½ = 0.301/-*m*, where *m* = the slope of the terminal phase of the plots. The times required to reduce virus burden to levels below an estimated human infectious dose_50_ (ID_50_, that is, the dose causing infections in 50% of those exposed) were calculated, assuming an initial viral burden of 1 × 10^6^ plaque-forming units (PFU). The times required to reduce the initial viral loads in the fabric by 1 log_10_ (*D*) were calculated by multiplying the terminal t½ values × 3.33 (one t½ = 0.301 log_10_ reduction in titer). The use of terminal half-life in such calculations in acknowledged to overestimate, to some extent, the times required for decay of the virus to levels lower than the ID_50_. These calculations, therefore, represent a more conservative approach than, for instance, calculations based on use of the initial t½ value or a calculated monophasic t½ value.

A human dose–response curve for SARS-CoV-2 has not yet been empirically determined, so an exact value for the human ID_50_ has not been reported. An ID_50_ of ~ 250 PFU was estimated, on the basis of mouse infectious dose_50_ values obtained for the betacoronaviruses mouse hepatitis virus (MHV-1)^22^ and SARS-CoV^23^. The time required to bring the fabric virus burden to 100 PFU (i.e., below the estimated ID_50_) was calculated as 4 log_10_ reduction × the time (*D*) required to achieve 1 log_10_ reduction in titer. This calculation was performed, as an illustrative example, to put the survival t½ data into perspective. It is acknowledged that the assumptions made were not based on empirical data in humans.

The human ID_50_ for influenza virus has been estimated, based on human volunteer studies, to be in the range of 0.6 to 3.0 TCID_50_, when administered in aerosols, and orders of magnitude higher when applied to the nasal mucosa^24^.

## Results

### Suspension virucidal efficacy testing

The results of testing of the virucidal efficacy of laundry sanitizers for viruses in suspension per EN 14476:2013 + A2:2019^12^ are displayed in Table 2. After a contact time of 15 min at a temperature of 20 ± 1 °C, the *p*-chloro-*m*-xylenol (PCMX)-based laundry sanitizer, at a final active concentration of 0.033% in hard water, resulted in > 5 log_10_ inactivation of both HCoV 229E and SARS-CoV-2. Under the same conditions, two quaternary ammonium compound (QAC)-based laundry sanitizers, tested at a final concentration of ~ 0.06% in hard water, resulted in ≥ 5 and > 4 log_10_ inactivation of SARS-CoV-2.

### Hard surface virucidal efficacy testing

The results of testing, per ASTM E1053-20^13^, of the virucidal efficacy of a QAC-based laundry sanitizer for coronaviruses and influenza viruses experimentally dried on a glass surface in the presence of a 5% fetal bovine sera (FBS) organic load are shown in Table 3. The results indicate complete inactivation (i.e., to the limit of detection of the assay) of each coronavirus and influenza virus following a 5-min contact time at 20 ± 1 °C. No lot-to-lot variability in virucidal efficacy was noted in these studies, which evaluated 2 to 3 independent product lots side-by-side under the same experimental conditions.

### Literature data on survival (persistence) of viruses on fabrics

Several studies of the survival (persistence of infectivity) of SARS-CoV-2 experimentally dried onto fabrics have been reported in the recent literature^6,15–19,25^. The data sets have been generated by determining infectious SARS-CoV-2 extracted from the fabric after various time periods following experimental contamination. The survival t½ values (times required to reduce the virus titer by one-half) were reported in the cited literature or were, in some cases^16,17,19^ calculated from reported raw data to reflect biphasic or monophasic decay values, as appropriate to the reported data sets. In some cases (e.g., the data of Virtanen et al.^25^), survival t½ values were not reported or able to be calculated from the reported data. Studies of the survival (persistence of infectivity) of influenza virus experimentally dried onto fabrics also have been reported^20,21^.

The viral persistence data are displayed in Table 4. We attempted to put the survival data into perspective by estimating the duration of time needed for the infectivity of the viruses to decay to levels lower than an estimated human ID_50_. Once fabrics are contaminated with SARS-CoV-2 or influenza viruses, these data suggest infectious virus may persist on the fabrics for minutes to days. While not displayed in Table 4, data on the persistence of SARS-CoV-2 and influenza H1N1 on non-porous or porous surfaces have been reviewed recently^26,27^.

**Table 4:** Literature values for terminal survival half-life (t½) of SARS-CoV-2 and influenza virus H1N1 on clothing/fabrics.

Prototypic fabric	Organic load	Temperature (RH)	Survival t½	Time needed for 1 log_10_ reduction in titer	Time needed to decrease viral burden below ID_50_^a^	Reference
**SARS-CoV-2**						
Cloth	None added	22 °C (65%)	27 min	1.5 h	5.9 h	^6^
Scrub (cotton/polyester)	None added	22 °C (40–50%)	1.0 h	3.3 h	13 h	^15^
Cotton cloths	None added	25–27 °C (35%)	23 h	77 h	306 h	^16^
Cotton	Tripartite soil	20 °C (35–40%)	1.7 h	5.7 h	23 h	^17^
Cotton	Tripartite soil	20 ± 1 °C (50%)	40 h	134 h	537 h	^18^
Cotton T-shirt	None added	21.5 ± 1 °C (45%)	10 h	34 h	136 h	^19^
Polyester sports shirt	None added	21.5 ± 1 °C (45%)	< 45 min	< 2.5 h	< 10 h	^19^
**Influenza virus H1N1**						
J-cloth	None added	17–21 °C (23–24%)	< 0.30 min	< 1 min	< 6.5 min	^20^
Jersey	None added	27 °C (37%)	1.3 min	4.3 min	28 min	^21^
Cardigan	None added	27 °C (37%)	2.9 min	9.5 min	62 min	^21^
T-shirt	None added	27 °C (37%)	3.5 min	12 min	78 min	^21^

*ID*_*50*_ infectious dose_50_, *RH* relative humidity, *t½* half-life; *tripartite soil* 0.25% bovine serum albumin, 0.35% tryptone, and 0.08% bovine mucin^29^.

^a^Calculated assuming an initial deposited virus burden of 1.0 × 10^6^ plaque-forming units (PFU) and an estimated human ID_50_ of 250 PFU (SARS-CoV-2); or an initial deposited virus burden of 1.0 × 10^6^ tissue culture infectious dose_50_ (TCID_50_) and an estimated human ID_50_ of 0.6 TCID_50_ (influenza virus).

## Discussion

The virucidal action of the clothes laundering process including drying in the electric dryer involves a combination of mechanical removal, microbicidal inactivation (detergent), and possible thermal inactivation. These occur even in the absence of added laundry-sanitizing agents. We are not suggesting or recommending, in the present article, that laundry sanitizers are required for sanitization of clothing contaminated by an enveloped virus. Laundry sanitizers may, however, be used during the pre-soak cycle to sanitize both the clothing articles being laundered, as well as the clothing-contact surfaces of the washing machine using targeted surface/hand hygiene agents (Fig. 1). There are other high-touch environmental surfaces (HITES) in the clothes-laundering area that are vulnerable to viral cross-contamination via the intermediacy of the launderer’s hands. These include appliance-operating knobs, clothes-folding surfaces, and even the operating controls and surfaces of drying appliances. The potential of virus dissemination to these primary and secondary surfaces (Fig. 1) may be mitigated, to some extent, by use of a laundry sanitizer capable of inactivating virus in wash solutions and dried on clothing-contact surfaces of the washing machine. However, a more holistic approach^10,11^ to interruption of viral dissemination during clothes laundering takes into account additional targeted interventions, such as surface and hand hygiene agents. Laundry sanitizers in combination with higher temperature may also be useful for enhancing the efficacy of the laundry process for inactivating non-enveloped viruses^10,11^, although that possibility has not been addressed in the current studies.

In the studies described here, we have employed both suspension and hard surface inactivation methodologies. The suspension method (BS EN 14476)^12^ was used to model the inactivation of virus in the wash and rinse solutions generated during clothes washing. Organic loads were employed in the testing to challenge the viral inactivation, although, in practice, any organic load associated with the virus would be expected to be greatly removed or diluted during the soaking, washing, and rinsing process. The hard surface method (ASTM 1053-20)^13^ involved drying of virus onto glass carriers to model inactivation of viruses dried on a hard, non-porous, surface, such as the metal tumbler of a washing machine, and transferred to and dried upon appliance door handles and operating knobs (Fig. 1). An organic load (5% FBS) was used to simulate the challenge associated with inactivating a virus dried in a soil matrix.

The standardized method ASTM E2274-16^28^, though appropriate for evaluating the efficacy of a laundry sanitizer, necessitates the use of a laundry tumbler. Such equipment is not normally available within a biosafety level 3 (BSL-3) laboratory such as that needed for working with highly pathogenic viruses, such as SARS-CoV-2.

The question of survival of infectious SARS-CoV-2 on fabric has been evaluated previously. The results, to date, are shown in Table 4, and have been put into perspective by relating the survival t½ data to possible initial viral burden and an estimated human ID_50_. SARS-CoV-2 RNA has been detected on fabric articles (pillow covers, duvet covers, sheets, and towels) taken from the quarantine hotel rooms of two patients three h after being tested positive for the virus^30^. Note that expected clothing/fabric SARS-CoV-2 burdens recoverable from naturally contaminated laundry items, in terms of infectious units, have yet to be empirically determined^31^, and this remains a knowledge gap. Similarly, the actual value of the ID_50_ for SARS-CoV-2 has yet to be determined^32^. Having said this, the data in Table 4 suggest that SARS-CoV-2 contamination on clothing may remain infectious for hours, and in the presence of a soil matrix, may remain infectious for days. Data for influenza viruses suggest that these also may remain infectious for hours on contaminated clothing.

Epidemiological, clinical, and laboratory evidence is accumulating^33^ that suggests that asymptomatic and pre-symptomatic SARS-CoV-2-positive patients shed infectious SARS-CoV-2 which can contaminate patient clothing, potentially cross-contaminating clothing of patient contacts and environmental HITES. Depending on the duration of time between contamination of a clothing article and laundering of the contaminated article, further contamination of the laundry appliance and the wash solutions with infectious virus is therefore possible. Manual (as opposed to machine) clothes washing, which still occurs to some extent even in developed countries, presents additional opportunities for contamination of secondary surfaces with infectious virus^34^. Infectious SARS-CoV-2 dried upon a hard surface (such as steel laundry tumbler) may remain infectious for days, based on a review of the survival data from the literature^26^. Similarly, data for survival of SARS-CoV-2 on skin^15,35^ indicate that the virus may remain infectious on contaminated skin for hours. The half-life of SARS-CoV-2 at 25 °C on human skin was found to be 3.5–4.2 h, while a half-life of 0.8 h was determined for influenza virus A^35^. Harbourt et al.^15^ reported that the half-life of SARS-CoV-2 on swine skin was 3.5 h at 22 °C. These survival data indicate that SARS-CoV-2 remains infectious on hard surfaces and human skin for hours to days, while influenza virus remains infectious for minutes to hours. This informs the need for hand and appliance hygiene practices to limit potential spread of virus (Fig. 1).

A recent study has indicated that SARS-CoV-2 can survive in wastewater, with a decay half-life of 0.49 d at ambient temperature^36^. These results are in agreement with empirical data indicating the persistence in wastewater of infectious mouse hepatitis virus-1 (a betacoronavirus), SARS-CoV (a betacoronavirus), and transmissible gastroenteritis virus (an alphacoronavirus)^37,38^, and for the alphacoronavirus HCoV-229E^39^. There is a possibility, therefore, of cross-contamination of otherwise virus-free clothing when washed together with a SARS-CoV-2-contaminated clothing article. Such possibilities could be mitigated through the use of an appropriately formulated laundry sanitizer with demonstrated efficacy for inactivating coronaviruses.

To suggest utility under field-use conditions, the concentrations of a formulated microbicidal active-containing laundry sanitizer tested in laboratory virucidal efficacy studies should be relevant to those obtained during clothes-washing when the laundry sanitizer is used as instructed. The QAC-containing products evaluated in Table 2 (suspension inactivation studies) are intended to be used in a pre-wash soak cycle (using a 1:42 dilution) for viral inactivation, relative to the concentration in the products themselves. The PCMX-containing product is intended to be used either in the wash cycle or in the pre-soak cycle. In either case, the product is recommended to be used a 1:50 dilution for 15 min contact time. The use concentrations and times have therefore been modeled appropriately in the suspension tests in Table 2. Under these conditions, inactivation of HCoV 229E or SARS-CoV-2 in the presence of soil load was complete, to the limit of detection of the assay used to determine titer. In all cases, > 4 log_10_ inactivation was observed. The products examined in hard surface inactivation studies (Table 3) were also very effective, causing ≥ 3 to ≥ 5.0 log_10_ inactivation of influenza viruses and coronaviruses, including SARS-CoV-2 in 5 min contact time.

## Conclusions

The risk of continued infectivity of virus on clothing/fabrics, once contaminated, is informed by survival data for those viruses on clothing, which suggest that virus may remain infectious for hours to days. There are multiple opportunities for dissemination of virus during the laundering process, and not all of these are addressed by the actual efficacy for removal and inactivation of the detergent and water-based washing and rinsing process. Other risks (Fig. 1) may best be mitigated through use of additional targeted hygiene agents, including possibly laundry sanitizers, surface hygiene agents, and hand hygiene agents.

Laundry sanitizers are used to enhance the efficacy of pathogen inactivation that may potentially occur during the manual or machine clothes washing and rinsing processes. A laundry sanitizer, added either during the pre-soak or wash stages of the washing process, may afford inactivation of viruses over that expected of the laundry detergent or hot water rinse alone, especially for non-enveloped viruses not expected to be inactivated by detergent^11^. In the case of the formulated microbicidal active-containing laundry sanitizing products evaluated in this study, the additional efficacy for inactivation afforded against the enveloped viruses SARS-CoV-2 and influenza virus amounted to ≥ 3 to ≥ 5 log_10_. These data suggest that use of a laundry sanitizer may afford additional mitigation of the risk of cross-contamination of the washing appliance (be it machine or basin), adjacent surfaces, the wastewater stream, and the hands of individuals engaging in washing of clothes contaminated with SARS-CoV-2, influenza viruses, or other emerging enveloped viruses.

## Data Availability

All data generated or analyzed during this study are included in this published article.
